# Externalizing behavior in early childhood and body mass index from age 2 to 12 years: longitudinal analyses of a prospective cohort study

**DOI:** 10.1186/1471-2431-10-49

**Published:** 2010-07-14

**Authors:** Sarah E Anderson, Xin He, Sarah Schoppe-Sullivan, Aviva Must

**Affiliations:** 1 Division of Epidemiology, The Ohio State University College of Public Health, Columbus, Ohio, USA; 2 Department of Epidemiology and Biostatistics, University of Maryland School of Public Health, College Park, Maryland, USA; 3 Department of Human Development and Family Science, The Ohio State University College of Education and Human Ecology, Columbus, Ohio, USA; 4 Department of Public Health and Community Medicine, Tufts University School of Medicine, Boston, Massachusetts, USA

## Abstract

**Background:**

Some evidence suggests that obesity and behavior problems are related in children, but studies have been conflicting and have rarely included children under age 4. An association between behavior problems in early childhood and risk for obesity could suggest that a common set of factors contribute to both. Our research objectives were to determine the extent to which externalizing behavior in early childhood is related to body mass index (BMI) in early childhood and through age 12, and to evaluate whether these associations differ by sex and race.

**Methods:**

Data from the NICHD Study of Early Child Care and Youth Development were analyzed. Externalizing behaviors at 24 months were assessed by mothers using the Child Behavior Checklist. BMI was calculated from measured height and weight assessed 7 times between age 2 and 12 years. Linear mixed effects models were used to assess associations between 24 month externalizing behavior and BMI from 2 to 12 years, calculate predicted differences in BMI, and evaluate effect modification.

**Results:**

Externalizing behavior at 24 months was associated with a higher BMI at 24 months and through age 12. Results from a linear mixed effects model, controlling for confounding variables and internalizing behavior, predicted a difference in BMI of approximately 3/4 of a unit at 24 months of age comparing children with high levels of externalizing behavior to children with low levels of externalizing behavior. There was some evidence of effect modification by race; among white children, the average BMI difference remained stable through age 12, but it doubled to 1.5 BMI units among children who were black or another race.

**Conclusions:**

Our analyses suggest that externalizing behaviors in early childhood are associated with children's weight status early in childhood and throughout the elementary school years, though the magnitude of the effect is modest.

## Background

The prevalence of obesity in very young children is high and has increased rapidly in the United States since the 1970 s [1,2]. Some evidence suggests that obesity is associated with child externalizing behavior problems [3-7], but an association has not been observed in all studies or in children of both sexes [5,8-11], and there is controversy regarding the magnitude [12] and directionality of any association [4,5,11], as well as the age when these associations might first be evident. Externalizing behavior problems are characterized by aggressive, oppositional, disruptive, or inattentive behaviors beyond those that would be expected given a child's age and development [13]. An association between behavior problems in early childhood and risk for obesity could suggest that a common set of factors contribute to both. Accumulating evidence indicates that the pathways in the brain governing appetite and emotion regulation are interrelated and may be impacted by stress [14].

Research has shown that children who experience maltreatment or neglect, bullying, social marginalization, or academic difficulties are more likely to experience externalizing behavior problems [15,16], and these factors have also been linked to obesity [17-20]. We found, in longitudinal analyses of the *Children in the Community *cohort, that disruptive behavior disorders identified prior to adolescence were associated with higher levels of relative weight and that these higher levels persisted into adulthood [7]. We also noted that this association appeared to be stronger among African American females [7]. The *Children in the Community *cohort includes individuals from New York State who were between 9 and 18 years of age in 1983, and their heights and weights were reported rather than measured. The findings from our previous analyses suggested the need to study associations between externalizing behavior and weight status in a diverse cohort observed from an early age, in which height and weight were measured repeatedly throughout childhood. Data collected during the *NICHD Study of Early Child Care and Youth Development (SECCYD) *provided this opportunity.

Through analyses of longitudinal data collected during the SECCYD, our objectives were to (1) determine the extent to which externalizing behavior in early childhood was related to children's body mass index (BMI) in early childhood and to their BMI trajectory through 12 years of age, (2) evaluate whether these associations differ by child sex and race, and (3) describe the magnitude of expected differences in BMI from age 2 to 12 years relative to children's externalizing behavior in early childhood with and without controlling for concurrent internalizing behavior.

## Methods

### Study Population

Data were collected during the *NICHD Study of Early Child Care and Youth Development *http://secc.rti.org. Briefly, the NICHD SECCYD was begun in 1991 with enrollment at 10 sites across the United States of 1,364 families at the birth of their healthy full-term infant. Participants were selected to ensure socioeconomic and ethnic diversity, and based on the mother's work intentions; exclusion criteria included mothers < 18 years, inability of the mother to communicate in English, intention of the family to move outside of the study area within 3 years, or the newborn having obvious disability or requiring a hospital stay of more than 1 week. Children and their families enrolled in the SECCYD have been followed from birth into adolescence through regular assessments that include direct observations and measurements, and personal interviews with parents, children, caregivers, and teachers, using procedures standardized across sites.

Primary hypotheses of the study concerned the influence of non-maternal care on children's developmental outcomes [21]. The study design and details of recruitment have been described in detail elsewhere [21]. Protocols were approved and reviewed annually by the institutional review boards of the participating universities. Data from the SECCYD are available to researchers by application. The Ohio State University has a data-use agreement for analysis of data from the SECCYD.

### Body mass index

Children's heights and weights were measured during laboratory visits at 24, 36, and 54 months, and when children were in the 1^st^, 3^rd^, 5^th^, & 6^th ^grades. A standardized protocol was used at all time periods and all sites. From height and weight, body mass index (BMI) was calculated (BMI = weight (kg)/height (m)^2^). Obesity was defined as a BMI-for-age above the 95^th ^percentile of the Centers for Disease Control and Prevention sex-specific BMI-for-age growth charts [22].

### Child externalizing and internalizing behavior

The Child Behavior Checklist (CBCL-2/3) was completed by mothers at their child's 24-month and 36-month laboratory study visit. The CBCL contains 100 items that assess whether the child, currently or in the past 2 months, has exhibited behaviors consistent with emotional or behavioral difficulties [23,24]. Mothers rated the extent to which each behavior described their child using the following three-level scale: "not true" (coded as 0), "somewhat true" (coded as 1), or "very true" (coded as 2). From these ratings six scales were derived: aggressive behavior (15 items), destructive behavior (11 items), anxious/depressed (11 items), withdrawn (14 items), sleep problems (7 items), and somatic problems (14 items). Age-normed scores (T scores) for externalizing behaviors and internalizing behaviors were calculated from the aggressive and destructive behavior scales (externalizing) and the anxious/depressed and withdrawn scales (internalizing). The CBCL has been used extensively and the reliability and validity of the instrument are well-established [13,24,25]. Our focus is on the association between externalizing behavior at 24 months and BMI trajectory from age 2 to age 12 years, but we also conducted sensitivity analyses using externalizing behavior assessed at 36 months. Our main analyses use the CBCL externalizing behavior T score as a continuous measure, but to determine whether covariates were associated with high levels of externalizing problems and assess confounding we categorized children as having high levels of externalizing behavior if their CBCL T score was ≥65 (this is the 95^th ^percentile of externalizing behavior at 24 months within the cohort, and is a cut point above which children's symptoms would be considered in the clinical range [23]).

### Covariates

Child age at each assessment was calculated based on date of birth and date of assessment. Birth weight (in grams) was recorded when children were enrolled in the study. The child's race was reported by mothers and we categorized children into two groups: white, and black or another race. Although Hispanic ethnicity was reported, too few children were Hispanic to allow for meaningful analysis. Mothers reported their educational attainment at the time of their child's birth. As a measure of financial resources at the 24 month assessment, we used an indicator for whether or not household income-to-needs ratio was less than the federal poverty threshold. Maternal depressive symptoms at 24 months were assessed using the Center for Epidemiological Studies Depression Scale (CES-D) and, as is customary, we considered a CES-D score > 16 to indicate probable depression [26].

### Analytic Approach

We included in analyses children who were not missing information on externalizing behavior at 24 months and whose BMI was assessed at least once at 24 months, 36 months, 54 months, 1^st ^grade, 3^rd ^grade, 5^th ^grade, or 6^th ^grade. All analyses were conducted using SAS v9.1 (SAS Institute, Cary, NC). Frequency distributions were compared using Chi-square tests or Fisher's exact test. We used logistic regression to estimate odds ratios and 95% confidence intervals for the cross-sectional association between obesity and high levels of externalizing behavior at 24 months overall and within strata defined by covariate levels. We used an alpha level of 0.05 to determine statistical significance in all analyses.

Linear mixed effects models [27] were used to estimate the average BMI trajectory and to test the extent to which externalizing behaviors at 24 months were related to differences in children's BMI. To determine the best shape for BMI trajectory relative to age we fit models with age as a linear, quadratic, and cubic term. We used a sequential process to build models predicting BMI relative to age centered at the cohort mean of 6.7 years. We tested whether externalizing behaviors at 24 months were related to average BMI and to linear change in BMI with age; we operationalized this by including externalizing behaviors at 24 months and the interaction of externalizing behaviors at 24 months and age in the linear mixed effects model. Evidence for an interaction between externalizing behaviors at 24 months and age would suggest that children's level of externalizing behavior at 24 months was related to BMI in a manner that depended upon age (for example, if there were no association between externalizing behavior and BMI at 24 months, but externalizing behavior at 24 months *was *associated with BMI at age 12 years).

We determined whether the association between BMI and externalizing behavior differed by sex and race by including cross-product terms in the model. Our final step in model building was to include covariates for possible confounding variables. Potential confounding variables we assessed were: sex, birth weight, maternal education, household poverty, and maternal depression. Potential confounding variables were retained in the model if their inclusion changed the coefficient for externalizing behaviors by more than 10% [28]. Internalizing behavior in early childhood could potentially confound any observed association between externalizing behavior and BMI and we assessed the effect on the association between externalizing behavior and BMI with and without inclusion of internalizing behavior at 24 months. We also conducted sensitivity analyses using externalizing behavior at 36 months, as well as the average of children's externalizing behaviors at 24 and 36 months.

To facilitate interpretation of model results we plot predicted BMI trajectories relative to high, medium, and low levels of externalizing behavior at 24 months. To further facilitate interpretation, we used contrast statements to estimate the predicted difference in BMI for two hypothetical children: one with high levels of externalizing behavior at 24 months (CBCL T score = 65) and the other with low levels of externalizing behavior at 24 months (CBCL T score = 35). We selected three illustrative time points (2 years, 7.5 years, and 11 years) to estimate predicted differences in BMI; these ages were selected to cover the age-range studied and at ages in which a majority of children were assessed.

## Results

Of 1,237 children with a measure of BMI at 1 or more assessments between 24 months and 6^th ^grade, 1,189 (> 95%) were not missing information on externalizing behaviors at 24 months and thus were included in our analyses. Table 1 presents characteristics of these children at each wave of assessment in which they were not missing BMI. The mean number of BMI measurements per child was 5.7 (SD = 1.8); 10% of the children had 3 or fewer, 50% had 6 or 7 out of a possible 7. Consistent with the United States population during this time period [29,30], the prevalence of obesity in the cohort rose from under 6% at 3 years of age to over 15% by age 9.

**Table 1:** Characteristics of participants included in analytic sample

	24 months	36 months	54 months	1^st ^grade	3^rd ^grade	5^th ^grade	6^th ^grade
n^a^	990	1055	1004	967	903	890	881
Mean age, years (SD)	2.1 (0.1)	3.1 (0.1)	4.7 (0.1)	7.0 (0.3)	9.0 (0.3)	11.0 (0.3)	11.9 (0.3)
Male, %	49.5%	50.5%	49.8%	49.8%	49.2%	50.3%	49.1%
White, %	82.0%	82.0%	82.8%	82.0%	81.9%	81.2%	81.5%
Mean height, cm (SD)	86.8 (3.2)	95.3 (3.5)	106.9 (4.3)	122.5 (5.4)	135.1 (6.3)	147.1 (7.3)	153.0 (7.6)
Mean weight, kg (SD)	12.7 (1.5)	14.7 (1.8)	18.4 (2.6)	25.4 (5.2)	34.0 (9.0)	44.0 (13.1)	49.2 (14.3)
Mean BMI (SD)	16.8 (1.4)	16.2 (1.4)	16.1 (1.6)	16.8 (2.6)	18.4 (3.8)	20.1 (4.8)	20.8 (4.9)
Obesity^b^, %	5.6%	5.9%	9.4%	11.9%	16.8%	19.2%	18.3%

SD = standard deviation, BMI = body mass index

^a ^Restricted to children not missing information on behavior problems at 24 months nor BMI at wave tabulated.

^b ^Obesity defined as BMI-for-age above the sex-specific 95^th ^percentile of the CDC BMI-for-age growth charts.

At 24 months, children's externalizing behavior T-scores ranged from a minimum of 30 to a maximum of 79; 6.2% of children had externalizing behavior scores at or above 65. Boys were slightly more likely than girls to have high levels of externalizing behavior; however the difference (7.0% vs 5.4%) was not statistically significant (p > 0.05). Race, maternal education, household poverty status, and maternal depression were all associated with differences in prevalence of high externalizing behavior at 24 months (Table 2). Cross-sectionally at 24 months, children with high levels of externalizing behavior had odds of obesity that were 2.9 (95% CI, 1.3, 6.5) times as high as children with lower levels of externalizing behavior (Table 2). Odds ratios for the association between high externalizing behavior and obesity at 24 months within strata of the covariates are also presented in Table 2.

**Table 2:** Prevalence and association of high externalizing behavior and obesity at 24 months relative to covariates

	n (%)	High externalizing behavior^a^: Prevalence (%)	Obesity^b^: Prevalence (%)	Odds ratio (95% confidence interval)^c^
Total	1189 (100%)^d^	74/1189 (6.2%)	55/990 (5.6%)	2.9 (1.3, 6.5)
**Sex**				
Boys	612 (51.5%)	43/612 (7.0%)	33/490 (6.7%)	2.7 (1.0, 7.6)
Girls	577 (48.5%)	31/577 (5.4%)	22/500 (4.4%)	2.7 (0.7, 10.2)
**Race**				
White	973 (81.8%)	50/973 (5.1%)*	39/812 (4.8%)*	1.7 (0.5, 5.8)
Black and Other	216 (18.2%)	24/216 (11.1%)	16/178 (9.0%)	3.6 (1.0, 12.4)
**Maternal education**^e^				
≥College degree	448 (37.7%)	11/448 (2.5%)*	12/378 (3.2%)*	- ^f^
Some college	402 (33.8%)	21/402 (5.2%)	20/341 (5.9%)	1.1 (0.1, 8.7)
≤High school graduate	339 (28.5%)	42/339 (12.4%)	23/271 (8.5%)	3.5 (1.3, 9.4)
**Household poverty status at 24 months**^g^				
≥poverty threshold	994 (84.8%)	44/994 (4.4%)*	38/835 (4.6%)*	3.3 (1.2, 8.9)
< poverty threshold	178 (15.2%)	26/178 (14.6%)	17/143 (11.9%)	1.6 (0.4, 6.2)
**Maternal depression at 24 months**^h^				
No	926 (83.2%)	38/926 (4.1%)*	41/776 (5.3%)	2.8 (0.9, 8.5)
Yes	187 (16.8%)	28/187 (15.0%)	10/152 (6.6%)	2.6 (0.6, 11.0)

*χ^2 ^or Fisher's exact test, *P *< 0.05; Fisher's exact test when expected cell frequency < 5.

^a ^High externalizing behavior defined as Child Behavior Checklist T score ≥ 65 at 24 months.

^b ^Obesity defined as BMI-for-age at or above the 95^th ^percentile of the sex-specific CDC BMI-for-age growth charts.

^c ^Odds ratio (95% Confidence Interval) from logistic regression models, predicting odds of obesity at 24 months with high externalizing behavior (≥ 65) at 24 months relative to lower levels of externalizing behavior (< 65).

^d ^Restricted to children who are not missing information on behavior problems at 24 months and who had 1 or more BMI measurements between 24 months and 6^th ^grade (n = 1189); 199 of these children were missing BMI at 24 months.

^e ^Maternal education at child's birth.

^f ^Unable to estimate within this strata due to data sparseness.

^g ^Seventeen with missing information excluded.

^h ^Maternal depression defined as CES-D score > 16; Seventy-six with missing information excluded.

Children's BMI trajectory from age 2 to 12 years was modeled as a cubic function of age. We tested whether the association between externalizing behaviors at 24 months and BMI trajectory differed for boys and girls; we saw no evidence for an interaction between sex and average BMI or change in BMI (p > 0.05) (results not shown). Externalizing behavior at 24 months was associated with average BMI trajectory (β = 0.21, p = 0.02), but there was little evidence overall that this association differed by age (β = 0.002, p = 0.38)(Table 3). We also tested whether associations differed by race (coded as a dichotomous variable: 0=white, 1=black or other); the interaction between race and externalizing behavior on change in BMI was statistically significant (p = 0.0002) (Table 3). We saw no evidence that child sex, birth weight, or maternal education confounded the association between externalizing behavior and BMI (results not shown). Maternal depression and household poverty at 24 months did result in changes of > 10% to the estimate for externalizing behavior and as a result were maintained in the final model. Adjustment for internalizing behavior at 24 months strengthened the association between externalizing behavior and BMI (Table 3). Models were repeated using externalizing and internalizing behavior problems assessed at 36 months, and the average of these behaviors at 24 and 36 months, and results were not substantively different (not shown). We also repeated analyses using sex-specific BMI z-scores based on the CDC BMI-for-age growth reference and observed consistent results (not shown).

**Table 3:** Association of externalizing behavior at 24 months with BMI from 24 months to 12 years^a^

	Association of 24 month externalizing behavior with BMI trajectory from 2 to 12 years	Including interaction between BMI, externalizing behavior and race	Adjusting for internalizing behavior
Race^b^	0.031 (0.11) P = 0.79	0.079 (0.65) P = 0.90	0.018 (0.65) P = 0.98
Ex^c^	0.021 (0.009) P = 0.02	0.015 (0.010) P = 0.14	0.026 (0.011) P = 0.02
Ex * age	0.002 (0.002) P = 0.38	0.00002 (0.002) P = 0.99	0.00002 (0.002) P = 0.99
Ex * race		0.011 (0.012) P = 0.37	0.013 (0.012) P = 0.31
Ex *age*race		0.003 (0.0007) P < 0.001	0.003 (0.0007) P < 0.001
Int^d^			-0.016 (0.006) P = 0.02

^a ^Coefficient (standard error) and P value from linear mixed effects models with random intercept and slope (age centered at mean of 6.7 years). All models are predicting BMI and include an intercept, linear, quadratic, and cubic effects of age, and adjustment for household poverty and maternal depression at 24 months. Sex, maternal education, and birth weight were evaluated as potential confounding variables and dropped from these final models. Externalizing and internalizing behaviors were modeled as continuous CBCL T-scores; model coefficients presented predict BMI changes associated with a 1 unit increase in 24 month CBCL T-score.

^b ^Race coded as 0=white, 1=black or other.

^c ^CBCL mother-reported externalizing behavior T score at 24 months.

^d ^CBCL mother-reported internalizing behavior T score at 24 months

**Figure 1:**
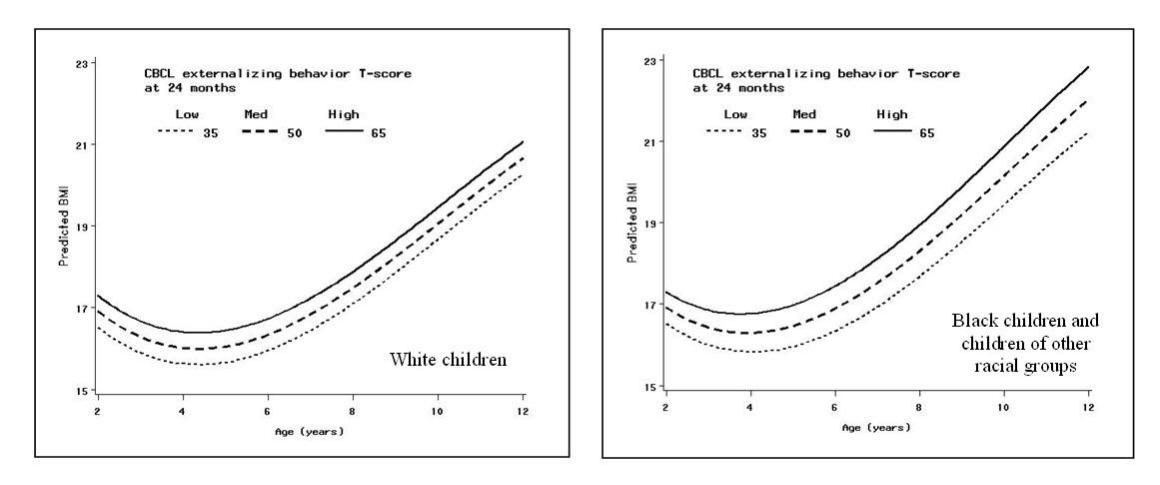
**Predicted BMI trajectories for low, medium, and high levels of externalizing behavior at 24 months**. Predicted BMI trajectories, by race, from final model in table 3 at three levels of externalizing behavior at 24 months: High (CBCL externalizing behavior T-score = 65, solid line), medium (CBCL externalizing behavior T-score = 50, heavy dashed line), and low (CBCL externalizing behavior T-score = 35, dotted line).

Figure 1 depicts predicted BMI trajectories by race from the final model in Table 3 at low, medium, and high levels of externalizing problems. The difference in BMI that would be predicted at 24 months for two white children who differed only in that one had a high level of externalizing behavior (CBCL T-score = 65) at 24 months and the other had a low level of externalizing behavior (CBCL T-score = 35) at 24 months is 0.78 BMI units (95% CI: 0.4, 1.2); at age 7.5 years the predicted difference in BMI between these 2 white children remains 0.78 BMI units (95% CI: 0.1, 1.5), and at age 11 years the predicted difference would still be 0.78 BMI units but the confidence interval is no longer statistically significant (95% CI: -0.3, 1.8). For a boy or girl of average height, an increase in BMI of 0.78 units corresponds to an increase of 1.3 pounds at age 24 months, 2.7 pounds at age 7.5 years and 3.6 pounds at age 11 years. For children who were black or of another race the predicted difference in BMI between a child with a high level of externalizing behavior at 24 months and a child with a low level of externalizing behavior at 24 months would be 0.77 BMI units (95% CI: 0.1, 1.5); at age 7.5 years the predicted difference in BMI between these 2 nonwhite children would be 1.23 BMI units (95% CI: 0.3, 2.1), and at age 11 years it would be 1.51 BMI units (95% CI: 0.3, 2.7). The corresponding difference in pounds for a child of average height would be 1.3 pounds at 24 months, 4.2 pounds at 7.5 years, and 6.9 pounds at 11 years.

## Discussion

Our results suggest that externalizing behavior problems are associated with higher BMI and obesity in children as young as 24 months old. Among two-year-old children, irrespective of race, based on our analyses we would predict an average difference of three-quarters of a BMI unit between children with high levels of externalizing behavior and children with low levels of externalizing behavior. This average difference in BMI was stable through age 12 among white children, and gradually doubled with age to about 1.5 BMI units among children who were black or of another race. These predicted average differences in BMI for a child with high levels of externalizing behavior compared to a child with low levels of externalizing behavior correspond to differences in weight of less than 2 pounds at 24 months of age, increasing to between 3 and 15 pounds at age 11 depending upon race and height.

It is well-established that weight status tends to track [31,32]; children who have a high BMI-for-age early in childhood are more likely than children with a low BMI-for-age to later be obese. Our analyses suggest that externalizing behaviors in early childhood are associated with children's weight status early in childhood and throughout the elementary school years, though the magnitude of the effect is modest. We are unaware of previously published analyses of externalizing behavior and weight status in children younger than age four.

A few studies of non-clinical populations have examined the relationship between obesity and externalizing behavior in preadolescent children and findings from these studies have not been consistent [3,5,6,8,11,12]. Some of the heterogeneity in results, summarized below, may be due to differences in assessment of externalizing behavior, analytic approach, and the population studied. In the *Longitudinal Study of Australian Children*, overweight 4- and 5-year-old children (born 1999-2000), had higher teacher-reported conduct problems than did non-overweight children, but differences were small [12]. In an earlier Australian cohort of children born 1981-1984, no association between behavior problems and obesity was observed for boys or girls cross-sectionally at age 5, however at age 14, compared to girls of normal weight status, girls who were overweight had over two times the odds of concurrent high levels of total behavior problems (externalizing behaviors were not examined separately); no association was seen for boys cross-sectionally at age 14 [11]. In a separate analysis of this same cohort, behavior problems at age 5 and age 14 years in both boys and girls were associated with higher BMI and obesity at age 21 years [4]. In the Netherlands, conduct problems were not related to overweight among 5- to 6-year-old children studied during 2004-2005 [8]. In the United States, evidence linking externalizing behavior problems in children to obesity is somewhat more consistent, with associations observed among 8- to 11-year-old children in the 1998 *National Longitudinal Survey of Youth *[3], and 5-year-old girls (but not boys) in the *Early Childhood Longitudinal Study - Kindergarten class 1998-1999 *[5]. In addition, oppositional defiant disorder was reported to be more prevalent among boys and girls in the *Great Smoky Mountain Study *(1993-2000) who were chronically obese between 9 and 16 years of age [6].

Using a different analytic approach than we have employed, Bradley *et al. *have also studied relationships between children's externalizing behavior, internalizing behavior, and BMI in the SECCYD [9]. They used cross-lagged structural equation models to address the directionality of associations between BMI and children's behavior. Our analyses of the same data were designed to address different research questions. We sought to understand the extent to which externalizing behavior early in childhood was associated with BMI early in childhood and change in BMI through age 12, to quantify the magnitude of any association, and to determine the extent to which associations were different in nonwhite children. The structural equation models used by Bradley *et al. *assume an association between BMI at 24 months, and behavior (externalizing and internalizing) at 24 months, and then controlling for that association and the tracking of BMI and behavior with age, ask whether BMI is associated with subsequent internalizing or externalizing behavior, and whether internalizing behavior or externalizing behavior are associated with subsequent BMI [9]. Although the results of the structural equation models they present do not allow for quantification of the magnitude of any associations, our findings and those of Bradley *et al. *are consistent. We found that externalizing behavior in early childhood was associated with higher weight status cross-sectionally, and that, among whites, the association between externalizing behavior at 24 months and BMI was maintained through childhood. This is consistent with Bradley *et al*.'s statement: "controlling for stability, ... externalizing behavior was not related to subsequent BMI" [9].

Our analyses provide evidence of a modest association between externalizing behavior early in childhood and BMI. We have quantified the magnitude of this association using linear mixed effects models, and also present odds ratios for the cross-sectional association at 24 months. We found that externalizing behavior and BMI were associated in two-year-old children. Our analysis does not allow us to identify whether the higher BMI came before, after, or developed concurrently with the higher levels of externalizing behavior. However *that *they are associated so early in children's lives suggests the need for further research. One might speculate that higher BMI could result from child behavior management techniques employed by parents and caregivers, such as use of food as a reward, to deal with challenges presented by toddlers with higher levels of externalizing behaviors. In an attempt to avoid a child's difficult or disruptive behavior, adults may acquiesce to demands for foods or activities (such as television viewing) associated with energy imbalance. We think the other direction of association - that externalizing behavior results from a child's high BMI - is less plausible. Perhaps more likely than either BMI or externalizing behavior being the cause or consequence of the other, is that a third factor or factors contributes to both a child's weight status/risk for obesity, and the degree to which they display externalizing behaviors. It is biologically plausible that such a factor or set of factors could impact both weight and behavior by influencing young children's brain development [14]. Identification of such modifiable risk or protective factors has potential to simultaneously help children achieve a healthy weight and decrease problematic behavior.

We provide evidence that the association between early childhood externalizing behaviors and BMI was not confounded by the sociodemographic covariates of sex, maternal education, or household poverty status, the association was as strong or stronger in children who were not white, and was not attenuated by adjustment for child internalizing behavior or maternal depression. We were not able to adjust for parental obesity, however, which is strongly related to child obesity and may be related to child behavior problems or maternal reports of child behavior problems. Our data suggest that the association between externalizing behavior and obesity at 24 months may differ by level of maternal education and household poverty status, in addition to race, but the pattern to these findings is not consistent, and further investigation is needed.

An additional limitation of our analyses is that we relied upon mother's reports of their child's behavior. The CBCL is not a diagnostic instrument and our analyses do not take into account the extent to which externalizing behaviors were problematic for the mother or the child. It is possible that ratings of children's behavior are influenced by the child's weight status. Considerable evidence indicates that obese children may be stigmatized and viewed negatively by peers and teachers [33], however the extent to which mother's reports of their toddler's externalizing behavior is influenced by weight status is not known.

## Conclusions

Although the degree to which BMI is elevated among children with high levels of externalizing behavior in early childhood is modest, in the context of the current prevalence of childhood obesity [1], understanding the factors that give rise to this association is important. Clinicians working in pediatric settings recognize that weight status and behavioral issues are topics frequently raised by parents of young children. Underlying family factors are likely related to both obesity and problematic child behavior, and the sensitivity of these topics for parents themselves compounds the hesitancy clinicians may have in addressing weight and behavior issues in children [34]. Continued research to elucidate these interrelationships in young children and families is needed and may lead to development of anticipatory guidance that is relevant and useful to parents.

## Abbreviations

BMI: Body mass index; CBCL: Child Behavior Checklist; CES-D: Center for Epidemiological Studies Depression Scale; CI: Confidence interval; NICHD: National Institute of Child Health and Human Development; OR: Odds ratio; SECCYD: Study of Early Childcare and Youth Development.

## Competing interests

The authors declare that they have no competing interests.

## Authors' contributions

SEA conceived and designed the study, conducted the analyses, and drafted the manuscript. XH participated in the design of the statistical analyses and interpretation of the models. SSS participated in the design of the study and interpretation of results. AM helped to conceive and design the study, participated in interpretation of results and helped to draft the manuscript. All authors read and approved the final manuscript.

## Pre-publication history

The pre-publication history for this paper can be accessed here:

http://www.biomedcentral.com/1471-2431/10/49/prepub
